# Supplementary material for: Vector borne disease control interventions in agricultural and irrigation areas in sub-Saharan Africa: A systematic review
Source: PLoS One. 2025 Feb 3;20(2):e0302279. doi: 10.1371/journal.pone.0302279 (PMC11790153; doi:10.1371/journal.pone.0302279)
Supplement: S1 Appendix — (DOCX) [file pone.0302279.s001.docx]

**S1 Appendix. Summary characteristics of excluded studies**

| **ID** | **Title** | **Rejection reason** |
| --- | --- | --- |
| Kibret S et al., 2017 | The Influence of Dams on Malaria Transmission in Sub-Saharan Africa | A Review |
| Hakizimana E- et al., 2022 | Community-Based Control of Malaria Vectors Using Bacillus thuringiensis var. Israelensis (Bti) in Rwanda | Focus was on the vector not VBD |
| Amin M et al., 2017 | Control of schistosomiasis in the Gezira irrigation scheme, Sudan | A Review |
| Ijumba JN et al., 2001 | Impact of irrigation on malaria in Africa: paddies paradox | Review article |
| Tusting LS et al., 2013 | Mosquito larval source management for controlling malaria | A Review |
| Ejezie GC et al., 1989 | The schistosomiasis problem in Nigeria | Policy brief |
| Kibret S et al., 2017 | Malaria impact of large dams at different eco-epidemiological settings in Ethiopia | No intervention was involved |
| Haileselassie W et al., 2021 | The effect of irrigation on malaria vector bionomics and transmission intensity in western Ethiopia | Study did not involve humans |
| Hawaria D et al., 2021 | Survivorship of Anopheles gambiae sensu lato in irrigated sugarcane plantation scheme in Ethiopia | Study did not involve humans |
| Paul P et al., 2018 | Land-use patterns and their implication on malaria transmission in Kilosa District, Tanzania | Irrigation not involved |
| Eba K et al., 2021 | Bio-Control of Anopheles Mosquito Larvae Using Invertebrate Predators to Support Human Health Programs in Ethiopia | Study did not involve humans |
| Abong RA et al., 2021 | The Mbam drainage system and onchocerciasis transmission post ivermectin mass drug administration (MDA) campaign, Cameroon | Study did not involve humans |
| Yewhalaw D et al., 2013 | The effect of dams and seasons on malaria incidence and anopheles abundance in Ethiopia | No intervention was involved |
| Mangani C et al., 2021 | Proximity of Residence to Irrigation Determines Malaria Risk and Anopheles Abundance at an Irrigated Agroecosystem in Malawi | No intervention was involved |
| Nyati-Jokomo Z et al., 2017 | Risk factors for schistosomiasis transmission among school children in Gwanda district, Zimbabwe | No intervention was involved |
| Kibret S et al., 2018 | Can water-level management reduce malaria mosquito abundance around large dams in sub-Saharan Africa? | Study did not involve humans |
| Yan G et al., 2022 | Impact of Environmental Modifications on the Ecology, Epidemiology, and Pathogenesis of Plasmodium falciparum and Plasmodium vivax Malaria in East Africa | Review |
| Cheke RA et al., 2023 | Seasonal variations and other changes in the geographical distributions of different cytospecies of the Simulium damnosum complex (Diptera: Simuliidae) in Togo and Benin | No intervention was involved |
| Sogoba N et al., 2007 | Malaria transmission dynamics in Niono, Mali: the effect of the irrigation systems | No intervention was involved |
| Muturi EJ et al., 2013 | Ecology and behavior of Anopheles arabiensis in relation to agricultural practices in central Kenya | No intervention was involved |
| Hawaria D et al., 2023 | Increased malaria incidence following irrigation practices in the Endorheic Rift Valley Basin of Sidama Region, Ethiopia | No intervention was involved |
| Spencer SA et al., 2021 | Five-Year Follow-Up on the Prevalence and Intensity of Infections of Schistosoma mansoni in a Hard-to-Reach District of Madagascar | Not irrigation area |
| Diuk-Wasser MA et al., 2007 | Effect of rice cultivation patterns on malaria vector abundance in rice-growing villages in Mali | Study did not involve humans |
| Kibret S et al., 2010 | The impact of a small-scale irrigation scheme on malaria transmission in Ziway area, Central Ethiopia | No intervention was involved |
| Rayaisse JB et al., 2015 | Baited-boats: an innovative way to control riverine tsetse, vectors of sleeping sickness in West Africa | Study did not involve humans |
| Dolo G et al., 2004 | Malaria transmission in relation to rice cultivation in the irrigated Sahel of Mali | Study did not involve humans |
| Fenwick A et al., 1970 | The development of snail control methods on an irrigated sugar-cane estate in northern Tanzania | Study did not involve humans |
| Alemayehu T et al., 1998 | Malaria, schistosomiasis, and intestinal helminths in relation to microdams in Tigray, northern Ethiopia | No intervention involved, just prevalence |
| Chikowore G et al., 2017 | A pilot study to delimit tsetse target populations in Zimbabwe | Study did not involve humans |
| Madsen H et al., 1986 | Trials on focal molluscicide application in larger irrigation canals and lakes in Mali | Did not involve humans |
| Ijumba JN et al., 2002 | Malaria transmission risk variations derived from different agricultural practices in an irrigated area of northern Tanzania | No intervention involved, just prevalence |
| Hawaria D et al., 2020 | Effects of environmental modification on the diversity and positivity of anopheline mosquito aquatic habitats at Arjo-Dedessa irrigation development site, Southwest Ethiopia | Study did not involve humans |
| Fenwick A et al., 1981 | The role of field irrigation canals in the transmission of Schistosoma mansoni in the Gezira Scheme, Sudan | Study did not involve humans |
| Djègbè I et al., 2020 | Minimal tillage and intermittent flooding farming systems show a potential reduction in the proliferation of Anopheles mosquito larvae in a rice field in Malanville, Northern Benin | Did not report on VBD reduction or increase |
| Muturi EJ et al., 2008 | Effect of rice cultivation on malaria transmission in central Kenya | Focus was on the vector not VBD |
| Ng'ang'a PN et al., 2008 | Malaria vector control practices in an irrigated rice agro-ecosystem in central Kenya and implications for malaria control | Focus was on the vector not VBD |
| Babiker A et al., 1985 | Focality and seasonality of Schistosoma mansoni transmission in the Gezira Irrigated Area, Sudan | Focus was on the vector not VBD |
| Meyer-Lassen J et al., 1994 | Evaluation of focal mollusciciding in the Rahad Irrigation Scheme, Sudan | Focus was on the vector not VBD |
| Omukunda E et al., 2012 | Effect of swamp cultivation on distribution of anopheline larval habitats in Western Kenya | Focus was on the vector not VBD |
| Fenwick A et al., 1972 | The costs and a cost-benefit analysis of an S. mansoni control programme on an irrigated sugar estate in northern Tanzania | Cost benefit analysis |
| Jones CM et al., 2023 | Integrating vector control within an emerging agricultural system in a region of climate vulnerability in southern Malawi: A focus on malaria, schistosomiasis, and arboviral diseases | Just lietrature review |
| Sturrock RF et al., 2001 | Seasonality in the transmission of schistosomiasis and in populations of its snail intermediate hosts in and around a sugar irrigation scheme at Richard Toll, Senegal | Focus was on the vector not VBD |
| Oladejo SO et al., 2006 | Unabated schistosomiasis transmission in Erinle River Dam, Osun State, Nigeria: evidence of neglect of environmental effects of development projects | No intervention involved |
| Zarroug IM et al., 2014 | The impact of Merowe Dam on Simulium hamedense vector of onchocerciasis in Abu Hamed focus - Northern Sudan | Did not involve humans |
| De Plaen R et al., 2003 | Living in the paddies: a social science perspective on how inland valley irrigated rice cultivation affects malaria in Northern Côte d'Ivoire | Focus was on the vector not VBD |
| Bolton P et al., 1988 | Schistosomiasis control in irrigation schemes in Zimbabwe | Not available |
| CROSSLAND NO, 1963 | A large-scale experiment in the control of aquatic snails by the use of molluscicides on a sugar estate in the northern region of Tanganyika | Focus was on the vector not VBD |
| MALEK EA et al., 1962 | Bilharziasis control in pump schemes near Khartoum, Sudan, and an evaluation of the efficacy of chemical and mechanical barriers | Focus was on the vector not VBD |
| Ijumba JN et al., 2002 | Irrigated crop production is associated with less malaria than traditional agricultural practices in Tanzania | No intervention involved |
| Khaemba BM et al., 1994 | Studies of anopheline mosquitoes transmitting malaria in a newly developed highland urban area: a case study of Moi University and its environs | Urban area |
| Highton RB et al., 1974 | The cost evaluation of mollusciciding operations on five irrigation schemes in Kenya | No abstract available |
| West PA et al., 2014 | Indoor residual spraying in combination with insecticide-treated nets compared to insecticide-treated nets alone for protection against malaria: a cluster randomised trial in Tanzania | Non irrigation area |
| Protopopoff N et al., 2015 | Combination of Insecticide Treated Nets and Indoor Residual Spraying in Northern Tanzania Provides Additional Reduction in Vector Population Density and Malaria Transmission Rates Compared to Insecticide Treated Nets Alone: A Randomised Control Trial | Non irrigation area |
| Bath D et al., 2021 | Effectiveness and cost-effectiveness of reactive, targeted indoor residual spraying for malaria control in low-transmission settings: a cluster-randomised, non-inferiority trial in South Africa | Non irrigation area |
| Afrane Y A et al., 2016 | Evaluation of long-lasting microbial larvicide for malaria vector control in Kenya | Non irrigation area |
| Mutero CM et al., 2020 | Evaluating the impact of larviciding with Bti and community education and mobilization as supplementary integrated vector management interventions for malaria control in Kenya and Ethiopia | Non irrigation area |
| Zhou G et al., 2013 | Modest additive effects of integrated vector control measures on malaria prevalence and transmission in western Kenya | Non irrigation area |
| Bousema T et al., 2016 | The impact of hotspot-targeted interventions on malaria transmission in Rachuonyo South District in the Western Kenyan Highlands: a cluster-randomized controlled trial | Non irrigation area |
| Kibret S et al., 2019 | Modeling reservoir management for malaria control in Ethiopia | Modelling study |
| Mahamat M. H. et al., | Adding tsetse control to medical activities contributes to decreasing transmission of sleeping sickness in the Mandoul focus (Chad) | Modelling study |
| Mazigo HD et al., 2019 | Malaria mosquito control in rice paddy farms using biolarvicide mixed with fertilizer in Tanzania: semi-field experiments | Did not target humans |
