# Supplementary material for: Vector borne disease control interventions in agricultural and irrigation areas in sub-Saharan Africa: A systematic review
Source: PLoS One. 2025 Feb 3;20(2):e0302279. doi: 10.1371/journal.pone.0302279 (PMC11790153; doi:10.1371/journal.pone.0302279)
Supplement: S2 Appendix — (DOCX) [file pone.0302279.s002.docx]

**S2 Appendix. Search strategies**

| Database | Strategy | # ref. |
| --- | --- | --- |
| PubMed | (((((IRS OR Indoor Residual Spray*, larvicid* OR ITNs OR Insecticide Treated Nets OR Long-lasting insecticide treated nets OR LLINs OR Nets OR molluscicide OR Mass drug administration OR health education OR Community awareness OR Community sensitization OR Training OR education OR Integrated Vector Management OR Pesticides OR Vector control) AND (Culicidae OR mosquito* OR Anopheles OR Aedes OR snails OR sand flies OR tsetse flies OR blackflies)) AND (malaria OR malaria transmission OR Plasmodium OR onchocerciasis OR schistosomiasis OR lymphatic filariasis OR dengue OR yellow fever OR chikungunya OR zika virus OR leishmaniasis OR sleep* sickness OR African trypanosomiasis)) AND (Irrigation OR Irrigation schemes OR dams OR wetlands)) AND (Central Africa OR Cameroon OR Central African Republic OR Chad OR Congo OR Democratic Republic of the Congo OR Equatorial Guinea OR Gabon OR Sao Tome and Principe OR Eastern Africa OR Burundi OR Djibouti OR Eritrea OR Ethiopia OR Kenya OR Rwanda OR Somalia OR South Sudan OR Sudan OR Tanzania OR Uganda OR Southern Africa OR Angola OR Botswana OR Eswatini OR Lesotho OR Malawi OR Mozambique OR Namibia OR South Africa OR Zambia OR Zimbabwe OR Western Africa OR Benin OR Burkina Faso OR Cabo Verde OR Cote d'Ivoire OR Gambia OR Ghana OR Guinea OR Guinea-Bissau OR Liberia OR Mali OR Mauritania OR Niger OR Nigeria OR Senegal OR Sierra Leone OR Togo OR Cape Verde OR Seychelles OR Comoros OR Republic of the Congo OR Congo Brazzaville OR The Gambia OR Madagascar OR Mauritius OR Western Sahara)) | 213 |
| Cochrane | (((((IRS OR Indoor Residual Spray*, larvicid* OR ITNs OR Insecticide Treated Nets OR Long-lasting insecticide treated nets OR LLINs OR Nets OR molluscicide OR Mass drug administration OR health education OR Community awareness OR Community sensitization OR Training OR education OR Integrated Vector Management OR Pesticides OR Vector control) AND (Culicidae OR mosquito* OR Anopheles OR Aedes OR snails OR sand flies OR tsetse flies OR blackflies)) AND (malaria OR malaria transmission OR Plasmodium OR onchocerciasis OR schistosomiasis OR lymphatic filariasis OR dengue OR yellow fever OR chikungunya OR zika virus OR leishmaniasis OR sleep* sickness OR African trypanosomiasis)) AND (Irrigation OR Irrigation schemes OR dams OR wetlands)) AND (Central Africa OR Cameroon OR Central African Republic OR Chad OR Congo OR Democratic Republic of the Congo OR Equatorial Guinea OR Gabon OR Sao Tome and Principe OR Eastern Africa OR Burundi OR Djibouti OR Eritrea OR Ethiopia OR Kenya OR Rwanda OR Somalia OR South Sudan OR Sudan OR Tanzania OR Uganda OR Southern Africa OR Angola OR Botswana OR Eswatini OR Lesotho OR Malawi OR Mozambique OR Namibia OR South Africa OR Zambia OR Zimbabwe OR Western Africa OR Benin OR Burkina Faso OR Cabo Verde OR Cote d'Ivoire OR Gambia OR Ghana OR Guinea OR Guinea-Bissau OR Liberia OR Mali OR Mauritania OR Niger OR Nigeria OR Senegal OR Sierra Leone OR Togo OR Cape Verde OR Seychelles OR Comoros OR Republic of the Congo OR Congo Brazzaville OR The Gambia OR Madagascar OR Mauritius OR Western Sahara)) in Title Abstract Keyword | 6 |
| African Index medicus | (tw:(Irrigation OR Farming OR Dam OR Wetlands OR Agricultural areas)) AND (tw:(IRS OR Indoor Residual Spray* OR larvicid* OR ITNs OR Insecticide Treated Nets OR Long-lasting insecticide-treated nets OR LLINs OR Nets OR Molluscicides OR Mass drug administration OR Health education OR Community awareness OR Community sensitization OR Training OR education OR Integrated Vector Management OR Pesticides OR Vector control)) AND (tw:(Routine OR Standard practice OR nothing)) AND (tw:(malaria OR malaria transmission OR Plasmodium OR onchocerciasis OR schistosomiasis OR lymphatic filariasis OR dengue OR yellow fever OR chikungunya OR zika virus OR leishmaniasis OR sleep* sickness OR African trypanosomiasis OR Malaria OR Bilharzia OR Schistosomiasis OR Dengue OR onchocerciasis OR Chikungunya OR Yellow fever OR African trypanosomiasis OR sleep* sickness OR leishmaniasis OR lymphatic filariasis OR Vector borne diseases)) AND (tw:(Algeria OR Angola OR Benin OR Botswana OR "Burkina Faso" OR Burundi OR Cameroon OR "Cape Verde" OR "Cabo Verde" OR "Central African Republic" OR Chad OR Comoros OR Comores OR Comoro OR Congo OR "Congo-Brazzaville" OR "Congo Republic" OR "Republic of the Congo" "Côte d'Ivoire" OR "Democratic Republic of the Congo" OR "DR Congo" OR DRC OR "Congo-Kinshasa" OR Djibouti OR "Equatorial Guinea" OR Eritrea OR Ethiopia OR Gabon OR Gambia OR "The Gambia" OR Ghana OR Guinea OR Guinea-Bissau OR Kenya OR Lesotho OR Liberia OR Madagascar OR Malawi OR Mali OR Mauritania OR Mauritius OR Mozambique OR Namibia OR Niger OR Nigeria OR Rwanda OR "Sao Tome and Principe" OR "São Tomé and Príncipe" OR Senegal OR Seychelles OR "Sierra Leone" OR Somalia OR "South Africa" OR "South Sudan" OR Sudan OR Swaziland OR Togo OR Uganda OR "United Republic of Tanzania" OR Tanzania OR Zambia OR Zimbabwe)) | 0 |
| Trip databse | irrigation OR farming OR dam OR wetlands OR agricultural areas, irs OR indoor residual spray* OR larvicid* OR itns OR insecticide treated nets OR long-lasting insecticide-treated nets OR llins OR nets OR molluscicides OR mass drug administration OR health education OR community awareness OR community sensitization OR training OR education OR integrated vector management OR pesticides OR vector control, routine OR standard practice OR nothing, malaria OR malaria transmission OR plasmodium OR onchocerciasis OR schistosomiasis OR lymphatic filariasis OR dengue OR yellow fever OR chikungunya OR zika virus OR leishmaniasis OR sleep* sickness OR african trypanosomiasis OR malaria OR bilharzia OR schistosomiasis OR dengue OR onchocerciasis OR chikungunya OR yellow fever OR african trypanosomiasis OR sleep* sickness OR leishmaniasis OR lymphatic filariasis OR vector borne diseases, Central Africa OR Cameroon OR Central African Republic OR Chad OR Congo OR Democratic Republic of the Congo OR Equatorial Guinea OR Gabon OR Sao Tome and Principe OR Eastern Africa OR Burundi OR Djibouti OR Eritrea OR Ethiopia OR Kenya OR Rwanda OR Somalia OR South Sudan OR Sudan OR Tanzania OR Uganda OR Southern Africa OR Angola OR Botswana OR Eswatini OR Lesotho OR Malawi OR Mozambique OR Namibia OR South Africa OR Zambia OR Zimbabwe OR Western Africa OR Benin OR Burkina Faso OR Cabo Verde OR Cote d'Ivoire OR Gambia OR Ghana OR Guinea OR Guinea-Bissau OR Liberia OR Mali OR Mauritania OR Niger OR Nigeria OR Senegal OR Sierra Leone OR Togo OR Cape Verde OR Seychelles OR Comoros OR Republic of the Congo OR Congo Brazzaville OR The Gambia OR Madagascar OR Mauritius OR Western Sahara  LMIC specific | 2696 |
| Google Scholar | irrigation OR farming OR dam OR wetlands OR agricultural areas, irs OR indoor residual spray* OR larvicid* OR itns OR insecticide treated nets OR long-lasting insecticide-treated nets OR llins OR nets OR molluscicides OR mass drug administration OR health education OR community awareness OR community sensitization OR training OR education OR integrated vector management OR pesticides OR vector control, routine OR standard practice OR nothing, malaria OR malaria transmission OR plasmodium OR onchocerciasis OR schistosomiasis OR lymphatic filariasis OR dengue OR yellow fever OR chikungunya OR zika virus OR leishmaniasis OR sleep* sickness OR african trypanosomiasis OR malaria OR bilharzia OR schistosomiasis OR dengue OR onchocerciasis OR chikungunya OR yellow fever OR african trypanosomiasis OR sleep* sickness OR leishmaniasis OR lymphatic filariasis OR vector borne diseases, Central Africa OR Cameroon OR Central African Republic OR Chad OR Congo OR Democratic Republic of the Congo OR Equatorial Guinea OR Gabon OR Sao Tome and Principe OR Eastern Africa OR Burundi OR Djibouti OR Eritrea OR Ethiopia OR Kenya OR Rwanda OR Somalia OR South Sudan OR Sudan OR Tanzania OR Uganda OR Southern Africa OR Angola OR Botswana OR Eswatini OR Lesotho OR Malawi OR Mozambique OR Namibia OR South Africa OR Zambia OR Zimbabwe OR Western Africa OR Benin OR Burkina Faso OR Cabo Verde OR Cote d'Ivoire OR Gambia OR Ghana OR Guinea OR Guinea-Bissau OR Liberia OR Mali OR Mauritania OR Niger OR Nigeria OR Senegal OR Sierra Leone OR Togo OR Cape Verde OR Seychelles OR Comoros OR Republic of the Congo OR Congo Brazzaville OR The Gambia OR Madagascar OR Mauritius OR Western Sahara | 4605 |
| Other sources (including references of other articles) |  | 248 |
| Total |  | **7768** |
