# Supplementary material for: Vector borne disease control interventions in agricultural and irrigation areas in sub-Saharan Africa: A systematic review
Source: PLoS One. 2025 Feb 3;20(2):e0302279. doi: 10.1371/journal.pone.0302279 (PMC11790153; doi:10.1371/journal.pone.0302279)
Supplement: Attachment — Submitted filename: Response to Reviewers.docx [file pone.0302279.s004.docx]

Dear Editor,

RESPONSE TO REVIEWERS

Below is a point-by-point response to the academic editor's and reviewer's comments. The responses are in italics.

**Reviewer’s comment number 1:**

Please ensure that your manuscript meets PLOS ONE’s style requirements, including those for file naming. The PLOS ONE style templates can be found at

https://journals.plos.org/plosone/s/file?id=wjVg/PLOSOne_formatting_sample_main_body.pdf and

https://journals.plos.org/plosone/s/file?id=ba62/PLOSOne_formatting_sample_title_authors_affiliations.pdf

**Response:**

*The title page has been edited accordingly.*

a. *Corresponding author indicated with an Asterisk (*), line number 3.*
b. *Author affiliations indicated with superscripts, lines 5 to 8.*

**Reviewer’s comment number 2:**

We note that the grant information you provided in the ‘Funding Information’ and ‘Financial Disclosure’ sections do not match.

When you resubmit, please ensure that you provide the correct grant numbers for the awards you received for your study in the Funding Information section.

**Response:** *Funding details updated: grant numbers verified.*

**Reviewer’s comment number 3:**

Thank you for stating the following in the Acknowledgments Section of your manuscript:

“We acknowledge NIHR funding to the study through the Shire-Vec Project.”

We note that you have provided funding information that is currently declared in your Funding Statement. However, funding information should not appear in the Acknowledgments section or other areas of your manuscript. We will only publish funding information present in the Funding Statement section of the online submission form.

Please remove any funding-related text from the manuscript and let us know how you would like to update your Funding Statement. Currently, your Funding Statement reads as follows:

“NIHR funded the study through the Shire-Vec Project.”

**Response:** *As instructed, the acknowledgement of financing has been removed from the manuscript. However, the acknowledgment should say as follows where it will be made;*

*“NIHR funded the study under grant number NIHR133144.”*

Please include your amended statements within your cover letter; we will change the online submission form on your behalf.

**Reviewer’s comment number 4:**

We notice that your supplementary tables are included in the manuscript file. Please remove them and upload them with the file type Supporting Information. Please ensure that each Supporting Information file has a legend listed in the manuscript after the references list.

**Response:** *Supplementary tables: Appendices S1 and S2 have been uploaded as supplementary documentation after being taken out of the manuscript.*

**Reviewer’s comment number 5:**

Please review your reference list to ensure that it is complete and correct. If you have cited papers that have been retracted, please include the rationale for doing so in the manuscript text, or remove these references and replace them with relevant current references. Any changes to the reference list should be mentioned in the rebuttal letter that accompanies your revised manuscript. If you need to cite a retracted article, indicate the article’s retracted status in the References list and also include a citation and full reference for the retraction notice.

**Response:** *Reference list verified; it is accurate.*

**Additional Editor Comments 6:**

Authors to go through and effect the corrections as stated

**Response:** *Reviewed the manuscript and made the necessary revisions.*

**Reviewers comment number 7 on Data Availability Statement:**

We note that your Data Availability Statement is currently as follows: [All relevant data are within the manuscript and its Supporting Information files.]

Please confirm at this time whether or not your submission contains all raw data required to replicate the results of your study. Authors must share the “minimal data set” for their submission. PLOS defines the minimal data set to consist of the data required to replicate all study findings reported in the article, as well as related metadata and methods (<https://journals.plos.org/plosone/s/data-availability#loc-minimal-data-set-definition>).

***Response:*** *I confirm that all the raw data required to replicate the results of your study (Qualitative) is within the manuscript and the supporting documents provided.*

Thank you.

Levi Kalitsilo (Corresponding Author).
